# Safety of cervical transforaminal epidural steroid injections

**DOI:** 10.1016/j.inpm.2024.100420

**Published:** 2024-06-05

**Authors:** William J. Beckworth, Gilad M. Ghanbari, Eduardo Lamas-Basulto, Benjamin Taylor

**Affiliations:** a AdventHealth, USA. 50 Hospital Drive, Hendersonville, NC, 28792, USA; b Department of Physical Medicine and Rehabilitation, Emory University, Atlanta, GA, USA; c Department of Anaesthesiology, Emory University, Atlanta, GA, USA

## Abstract

**Background:**

In 2014 the FDA issued a drug safety warning that steroids in the epidural space may result in rare but serious neurological adverse events. The FDA identified 131 cases of neurological adverse events and most complications were related to cervical transforaminal epidural injections (TFESIs). These complications occurred before the standard use of non-particulate steroids. Many still consider cervical TFSEIs to be unsafe.

**Objectives:**

The objective of this study was to evaluate the safety of cervical TFESIs with non-particulate steroids.

**Methods:**

A review was done of all cervical TFESIs from 2004 to 2021 at an academic institution when non-particulate steroids became more commonly used by reviewing CPT code 64479 linked to the performing physician. All treating physicians and department directors were queried about catastrophic complications (stroke, spinal cord injury, death or other). A secondary analysis was done on 200 consecutive cervical TFESIs looking at immediate and delayed side-effects documented by the nurse in recovery, day-after phone calls and clinic follow-up notes.

**Results:**

From 2004 to 2021 the CPT code 64479 was used 6967 times, with 6241 cervical TFESIs and 726 thoracic TFESIs. No catastrophic complications occurred. In the subset analysis of 200 consecutive cervical TFESIs, 7 patients (3.5 %, 95 % CI 1.0–6.0) had a transient increase in pain, 18 (9 %, 95 % CI 5.0–13.0) had no change in pain and 171 (85.5 %, 95 % CI 80.6–90.4) had a decrease in pain. The average pain score among all participants dropped 3.7 (95 % 3.0–4.4) points. A 2-point drop was seen in 75.5 % (95 % CI 69.5–81.5) and a 3-point drop was seen in 62.5 % (95 % CI 59.1–65.9). Five of the seven patients with transient increased pain had an increase of ≥ 3 points on numerical rating scale. There was one of each of the following reported: insomnia, glucose >500, transient thumb numbness with pain, and hypertension. Two cases of headaches were reported.

**Conclusion:**

This study supports the safety of cervical TFESIs with non-particulate steroids as recommended by consensus opinions from medical societies.

## 1 Introduction

Neck pain and cervical radicular pain are common pathologies. Globally, the point prevalence of neck pain with or without radicular symptoms was reported at 3.5 % or specifically in 3551 per 100,000 individuals [1]. In the United States, the prevalence of cervical radicular pain was 83 per 100,000 individuals [2]. The most common cause of cervical radicular pain is a disc herniation or cervical spondylosis causing cervical nerve root irritation [3]. Although the natural history is generally favorable, pain can be quite debilitating with a subset of patients needing surgery [4]. The conservative management includes cervical epidural injections.

Cervical epidurals are generally safe, but catastrophic complications have been reported with both interlaminar and transforaminal epidural injections [5]. In 2014 the FDA issued a drug safety warning that steroids in the epidural space may result in rare but serious neurological adverse events. The FDA identified 131 cases of neurological adverse events and the majority of complications were related to cervical transforaminal epidural injections (TFESIs) [6]. These complications came before the standard use of non-particulate steroids.

In the early 2000s studies linked intra-arterial injections of particulate steroids as a likely cause of embolic infarcts to the spinal cord and brain. A study in 2004 looked at common steroid preparations under a light microscope and found that particulate steroids tended to aggregate or clump into a size that could be a source of an embolic infarct [7]. Another study looked at injection of particulate steroids versus non-particular steroids into the vertebral artery of pigs. The animals injected with a particulate steroid never regained consciousness. MRI and histological evaluation also showed upper cervical cord and brainstem edema and ischemia in those injected with a particulate steroid. None of these findings were seen when a non-particulate steroid was used [8].

The consensus recommendation from multiple societies is to use non-particulate steroids for cervical TFESIs [9]. With the use of non-particulate steroids, no catastrophic complications have been reported. Yet many providers still consider cervical TFESIs to be unsafe. The purpose of this study is to look at the safety of cervical TFESIs with the use of non-particulate steroids.

## 2 Methods

After obtaining institutional review board (IRB) approval, a review was done on all cervical TFESIs done at an academic institution between 2004 and 2021. In the mid 2000s, non-particulate became more commonly used at this institution. The electronic medical record system Cerner (Oracle Cerner, Oracle Health) was searched to retrieve all procedures with the CPT (current procedural terminology) code 64479. Additional information was obtained including the description of the procedure, performing physician and J-code. Since CPT code 64479 is inclusive of both cervical and thoracic TFESIs, the description of the procedure differentiated cervical from thoracic TFESIs. J-codes were used to document what steroid was used.

Each procedure was linked to the performing physician. Those physicians were directly contacted and queried about catastrophic complications such as spinal cord injury, stroke, death, infection, or any other concerning event. Additionally, department directors for both anesthesia pain and physical medicine and rehabilitation (PM&R) were also asked about any catastrophic complications among their physicians.

The second part of the study was a limited review of two hundred consecutive cervical TFESIs looking at immediate and delayed side effects or complications that were more commonly seen. These more common side effects or complications could not be adequately retrieved on all procedures from 2004 to 2021, thus a more focused chart review was done on a smaller consecutive group of patients.

This limited review involved looking at nursing documentation in recovery (immediate post-procedure), nurse call-back one day after procedure, clinic follow-up notes and urgent clinic visits (ER, urgent care, primary care). The nursing notes in recovery documented pre- and post-procedural pain, vitals, medications given and any issues that occurred while in recovery. The nurse call-back was done one day after the procedure as a standard practice to follow up and document patient status. Lastly, the follow-up clinic notes and urgent visits (ER, urgent care, primary care) were reviewed for complications. Clinic follow-up intervals varied by provider.

Most cervical TFESIs were performed by physicians with PM&R and anesthesia backgrounds and minority were done by radiologists. While there was a small variability in procedural techniques, all procedures performed by PM&R ad anesthesia physicians followed the guidelines for cervical TFESIs as outlined by the International Pain and Spine Intervention Society [10]. Standard practice included fluoroscopic guidance, oblique approach, needle placement in the posterior neural foramen, 25-gauge needle, extension tubing and contrast administration with live fluoroscopy. Digital subtraction angiography was available during this time frame and used by some. Lastly, while non-particulate steroids were more commonly used from the mid-2000s onward, this was not universally utilized early on by all providers.

## 3 Results

The CPT code 64479 was used 6967 times between 2004 and 2021. This included 6241 cervical TFESIs and 726 thoracic TFESIs. No catastrophic complications (spinal cord injury, stroke, death or other) were reported by providers or directors of the anesthesia pain and PM&R departments.

Of the 6967 procedures done only 34 procedures were by providers that were not known or could not be contacted. This means the known providers accounted for 6933 of the 6967 total procedures. Known providers were queried as were their department directors.

The physicians doing these procedures included 16 PM&R physicians, eleven anesthesiology pain physicians, six radiologists and one orthopaedist. The 6241 cervical TFESIs were completed by PM&R (5791 procedures), anesthesiology pain (432 procedures), radiology (17 procedures) and orthopaedics (1 procedure). The 726 thoracic TFESIs were completed by PM&R (681 procedures), anesthesiology pain (41 procedures) and radiology (4 procedures). Only a small percentage of J-codes were able to be retrieved and thus the exact steroid used was unable to be verified.

The second part of the study captured two hundred consecutive cases and looked at milder side effects and complications. A total of seven patients or 3.5 % (95 % CI 0.95–6.0) reported an increase in pain immediately after the procedure. Five of the two hundred, or 2.5 % (95 % CI 0.34–4.7), reported an increase of ≥3/10 pain on a numerical rating scale (NRS). One case went to urgent care after the procedure for pain. A total of eighteen patients or 9 % (95 % CI 5.0–13.0) reported no change in pain and 171 patients or 85.5 % (95 % CI 83.0–88.0) reported a decrease in pain. The mean drop in pain score was 3.7 points post-procedure. Categorical data of at least a 2-point drop was seen in 75.5 % (95 % CI 69.5–81.5) and a drop of at least 3-points was seen in 62.5 % (95 % CI 59.1–65.9) of patients.

In the two hundred consecutive patients other complications beyond pain were captured. There was one case of increased “muscle spasms” reported. There was one case of insomnia and one case of a headache which was not further differentiated. One patient had an increase in blood glucose greater than 500. There was one case of transient numbness and pain to the thumb. One patient had elevated blood pressure after the procedure. Lastly, there was one report of a rash to the right side of the neck where the procedure was done (see Table 1).

**Table 1:** Immediate complications after cervical transforaminal epidural steroid injections.

Immediate Complications	Number	Percentage with 95 % CI
Increase in pain	7/200	3.5 % (0.95–6.0)
Increase of pian ≥3/10	5/200	2.5 % (0.34–4.7)
Increase pain requiring urgent care	1/200	0.5 % (0.0–1.5)
Muscle spasm	1/200	0.5 % (0.0–1.5)
Insomnia	1/200	0.5 % (0.0–1.5)
Elevated glucose (>500)	1/200	0.5 % (0.0–1.5)
Transient numbness thumb	1/200	0.5 % (0.0–1.5)
Elevated blood pressure	1/200	0.5 % (0.0–1.5)
Neck rash	1/200	0.5 % (0.0–1.5)

## 4 Discussion

This study supports the safety of cervical TFESIs when non-particulate steroids are used. To our knowledge this is the largest series of cervical TFESIs reviewing safety and complications. This data supports the consensus opinion from a multidisciplinary working group and national organizations which state that “the ultimate choice of what approach or technique (interlaminar vs transforaminal epidural steroid injection) to use should be made by the treating physician by balancing potential risks vs benefits with each technique for each given patient” [9].

In 2014 the FDA issued a drug safety warning about rare but serious neurological adverse events with epidurals, with the majority related to cervical TFESIs. These case reports were primarily associated with particulate steroid use. To the knowledge of the authors, there have been no case reports of catastrophic complications with a cervical TFESIs when a non-particulate steroid (dexamethasone) was used. There was one case report of a spinal cord injury with a cervical TFESI with 4 mL volume of dexamethasone, hyaluronidase and mepivacaine but this was secondary to an unfortunate direct injection into the spinal cord [11]. There was one case report of a right L4 TFESI with dexamethasone with a resulting conus medullaris infarct which does raise concern. Yet this was a single case report of uncertain etiology among the millions of epidurals done each year [12].

Cervical ILESI are considered by some as a “safer” procedure compared to cervical TFESIs, but this may not be true. In a 2011 study reviewing malpractice claims over a three-year period from the ASA's (American Society of Anesthesiology) closed claims database, most of the cervical epidural malpractice claims were related to cervical ILESIs. There were twenty-seven cases of spinal cord injury (twenty from ILESI and ten from TFESI) and nine cases of non-spinal cord injury (seven from ILESI and two from TFESI). Thus, twenty-seven complications were attributed to cervical ILESIs and twelve to cervical TFESIs [13]. It is likely that more ILESIs were done and that may partially account for the increased number of complications with cervical ILESIs vs TFESIs. Yet cervical TFESI complications were primarily attributed to particulate steroid use. Had non-particulates been used, complications with TFESIs may have been less.

The 2011 ASA malpractice claims paper highlights that cervical ILESIs can have catastrophic complications. There were 64 malpractice cases associated with cervical procedures with 51 of those being procedure adverse events and 13 non-procedure issues (incorrect diagnosis, expectations, positioning, fall etc.). Of the 51 procedure complications, cervical ILESIs caused the majority. Complications included 20 with needle trauma to cord/nerve, 4 dural punctures, 3 hematomas with cord compression, 3 infections/abscesses and 3 high total spinal blocks. These accounted for 33 of the 51 cases (64.7 %) and were primarily attributed to cervical ILESIs. Whereas there were nine cases (9/51 or 17.6 % of cases) from cervical TFESI related to embolic infarcts [13] which may have been prevented with a non-particulate steroid.

Many safety measures for cervical ILESIs can be employed to prevent complications. This includes reviewing MRI, limiting sedation, utilizing image guidance and staying at or below C6-7 (preferably at C7-1). The epidural space is normally less than 2–3 mm at C7-T1 for ILESIs [10] and in very close proximity to the spinal cord. It is known that there are gaps in the ligamentum flavum that are consistently located in the midline which can impair loss-of-resistance techniques. Gaps are seen between 51 and 71 % of the time at C7-T1 [14,15]. Another study reported an incidence of midline gaps in the ligamentum flavum from C3-T2 ranging from 87 to 100 % [16]. Thus, a paramedian approach is probably safer at C7-T1. While most complications with cervical ILESIs may be prevented, certain complications (dural puncture, hematoma, cord compression) could still be encountered.

Safety measures can be employed to avoid complications with cervical TFESIs. Most important is using a non-particulate steroid. Animal studies have shown that even if injected into the vertebral artery no embolic stroke or spinal cord injury occurs [8]. Reviewing axial T2 weighted images on MRI before the procedure is recommended to look at the location of the vertebral artery which can be occasionally located posteriorly in the neural foramen [17]. Multiplanar imaging along with live contrast injection to visualize vascular flow in the spinal radiculomedullary artery or vertebral artery is essential. It is also important to use extension tubing to keep the needle still. Using digital subtraction angiography (DSA) is an optional method which is more sensitive at picking up vascular flow [18]. There is also the option of using a test dose of local anesthetic.

A test dose of local anesthetic can be helpful in detecting vascular uptake that is undetected by other routes [19]. This can especially be helpful with the radiculomedullary artery which can be difficult to consistently visualize on fluoroscopy. Symptoms elicited from positive test dose include things such as metallic taste, ringing in ears, tachycardia, dizziness, wide spread paresthsia or paresis. One striking example of this was a case of quadriplegia after a test dose of local anesthetic. The case was aborted and the quadriplegia resolved after 20 min [20]. Thus, a test dose can be a helpful tool but with the use of dexamethasone may be considered optional.

The modified approach for a cervical TFESI has also been recommended as a safer approach which helps avoid vessels such as the vertebral artery and internal carotid artery [21]. This modified approach is parallel to the ventral surface of the superior articular process and is generally between 66 and 72° oblique from midline as opposed to the traditional approach (parallel to the lamina) which is around 50° oblique from midline [21]. Additionally, the modified approach appears to lead to better epidural flow [22]. It should be noted that about 50 % of the time contrast will flow around the vertebral artery (extravascular) with either approach. Lastly, there is the possibility to encounter the brachial plexus as the needle approaches the foramen for a TFESI. The more oblique approach (modified approach) may be less likely to encounter the brachial plexus.

There are weaknesses with this study. It is a retrospective review of the electronic medical record system. The lack of J codes makes it impossible to verify non-particulate steroid use in cervical TFESIs. Dexamethasone became more commonly used at this institution during the mid 2000s, yet it was not exclusively used in the early years of this study.

There are other weaknesses with this study. This study relied on the recall of physicians who may be unwilling to report catastrophic complications. Department directors were queried as well about significant complications to counter this issue. The second part of this study relied on chart documentation which has inherent limitations. There is the chance for underreporting of side effects and complications in clinic documentation. There are issues with follow-up intervals in this study. While there was consistency with the nurse phone call a day after each procedure, the time frame for clinic visits were inconsistent.

## 5 Conclusion

This study supports the safety of cervical TFESIs with non-particulate steroids as recommended by consensus opinions from medical societies.

## Funding

None.

## Declaration of competing interest

The authors declare the following financial interests/personal relationships which may be considered as potential competing interests:

William Beckworth reports a relationship with FusMobile that includes: funding grants. If there are other authors, they declare that they have no known competing financial interests or personal relationships that could have appeared to influence the work reported in this paper.
